# Abdominal obesity increases metabolic risk factors in non-obese adults: a Hungarian cross-sectional study

**DOI:** 10.1186/s12889-019-7839-1

**Published:** 2019-11-15

**Authors:** Anita Lukács, Edina Horváth, Zsuzsanna Máté, Andrea Szabó, Katalin Virág, Magor Papp, János Sándor, Róza Ádány, Edit Paulik

**Affiliations:** 1 Department of Public Health, Faculty of Medicine, University of Szeged, Dóm tér 10, Szeged, 6720 Hungary; 2 Department of Medical Physics and Informatics, University of Szeged, Szeged, Hungary; 3 National Public Health Center, Budapest, Hungary; 4 Department of Preventive Medicine, Faculty of Public Health, University of Debrecen, Debrecen, Hungary; 5 MTA-DE Public Health Research Group, Department of Preventive Medicine, Faculty of Public Health, University of Debrecen, Debrecen, Hungary

**Keywords:** Abdominal obesity, Waist circumference, Metabolic syndrome, Prevention, Screening

## Abstract

**Background:**

The prevalence of abdominal obesity is increasing worldwide. Adults with abdominal obesity have been reported to have increased risk of cardiometabolic disorders.

The aim of this study was to examine whether non-obese subjects (body mass index (BMI) < 25 kg/m^2^) with abdominal obesity examined in the framework of the Swiss–Hungarian Cooperation Programme had increased metabolic risk compared to participants without abdominal obesity.

**Methods:**

A cross-sectional study was carried out in 5228 non-obese individuals. Data were collected between July 2012 and February 2016. Descriptive statistics, Pearson’s correlation analysis and multiple logistic regression models were applied, odds ratios (OR) with 95% confidence interval (CI) being the outcomes.

**Results:**

607 (11.6%) out of the 5228 non-obese individuals had abdominal obesity. The correlation analysis indicated that the correlation coefficients between BMI and waist circumference (WC) were 0.610 in males and 0.526 in females. In this subgroup, the prevalence of high systolic blood pressure, high fasting blood glucose, and high total cholesterol and triglyceride levels were significantly higher. The logistic regression model based on these data showed significantly higher risk for developing high systolic blood pressure (OR = 1.53; 95% CI = 1.20–1.94), low HDL cholesterol (OR = 2.06; 95% CI = 1.09–3.89), and high trygliceride level (OR = 1.65; 95% CI = 1.27–2.16).

**Conclusions:**

There was a very high, significant, positive correlation between WC and BMI. Abdominal obesity was found to be strongly related to certain metabolic risk factors among non-obese subjects. Hence, measuring waist circumference could be recommended as a simple and efficient tool for screening abdominal obesity and related metabolic risk even in non-obese individuals.

## Background

Obesity is one of the major public health concerns of our era: its prevalence has increased significantly in the past decades both globally [1, 2] and in Hungary [3, 4].

In Hungary, many people live with a high risk of metabolic syndrome, which promotes the development of atherosclerotic vascular diseases and type 2 diabetes mellitus (T2DM) [5], mainly due to abdominal obesity. The prevalence of the latter was 38% in males and 55% in females [6] in 2014, and the morbidity of T2DM has continuously been increasing [7]. Therefore, it is essential for heath professionals to be aware of the metabolic risk of this population.

Body mass index (BMI) is widely used to monitor the prevalence of obesity because the risk of coronary heart disease, ischemic stroke, and T2DM rises in parallel with the increase in the BMI [8]. Unfortunately, BMI provides limited information about body fat content and no information on central fat distribution, the degree of which may be more closely related to metabolic risks than BMI itself [9]. Body adiposity varies according to age and gender, and BMI alone is not appropriate to distinguish between persons with excess body fat and persons with high muscle mass, to whom the same metabolic risk would be attributed based purely on their BMI [10].

Nonetheless, several reports suggest that the measurement of waist circumference (WC) can compensate the above described limitation of BMI since WC is more closely related to visceral fat content, and thus to metabolic risks. Previous research shows that on the one hand, normal weight individuals with abdominal obesity can have metabolic risk factors, and therefore being candidates for having elevated risk for metabolic syndrome [11] or cardiovascular diseases (CVDs) [12], and on the other hand, measurements of abdominal obesity, principally WC, are more closely related to metabolic risk factors than the index of general adiposity [13]. According to Park et al. [10], WC as a marker of obesity is a better predictor of coronary artery calcification than BMI. Subjects with abdominal obesity were more likely to have a metabolic syndrome compared to those without abdominal obesity, therefore, WC should also be measured and used in conjunction with BMI to assess and predict metabolic risk, where possible, according to the conclusion of a recent report of the World Health Organization (WHO) [8].

There is, however, limited information about the usefulness of WC measurement in identifying cardiometabolic risk factors in a large study sample. Therefore, in our study, we aimed to examine whether non-obese subjects with no chronic conditions but abdominal obesity had a higher chance of having related metabolic risk factors, such as elevated blood pressure, high fasting blood glucose, high cholesterol, high low-density lipoprotein (LDL), low high-density lipoprotein (HDL), and high triglyceride levels, compared to persons with no abdominal obesity.

## Materials and Methods

### Study design and subjects

The programme, named ‘Public Health Focused Model Programme for Organising Primary Care Services Backed by a Virtual Care Service Centre’ (the Programme), has been an innovative health promotion and public health project, launched in July 2012 in Hungary in Swiss–Hungarian cooperation. The aim of the Programme was to provide efficient preventive services, contributing thereby to the improvement of the general health status of the population, and the reduction of social health inequality [14]. An element of the new services developed by the programme was the general practitioners’ cluster (GPC) model (described elsewhere [15, 16]), which was designed to establish a community-oriented screening system in Hungary with the aim of providing comprehensive evaluation of the health status of all adults above 18 years of age belonging to the GPCs, irrespective of their health status. The health check surveyed the sociodemographic status, lifestyle and health attitude (nutrition, physical activity, alcohol use, smoking), mental health, and history of chronic diseases and screenings for cardiometabolic risk factors and hidden diseases. The Health Status Assessment (HSA) was implemented by a team of a trained nurse and a trained public health practitioner [17], the task of who was to recruit the subjects, collect questionnaire-based data, and perform the physical and laboratory examinations.

**Fig. 1:**
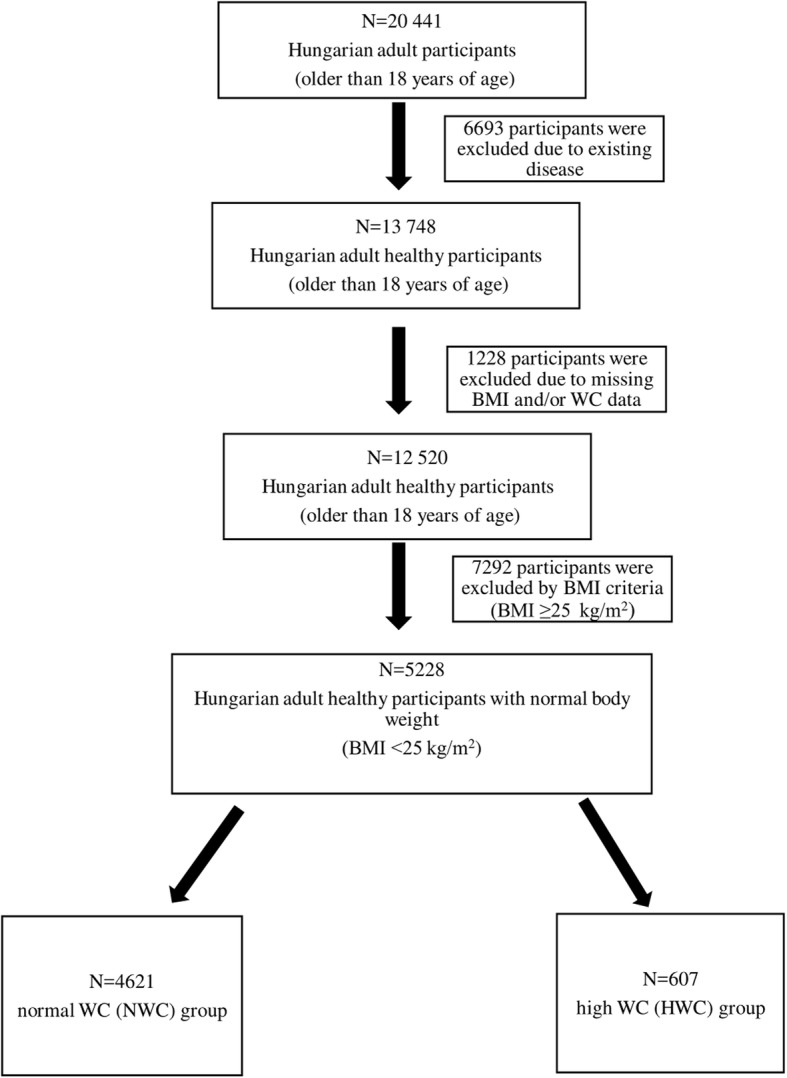
Schematic diagram of the study sample. BMI: body mass index, WC: waist circumference

It was a cross-sectional study conducted in adult persons taking part in the Programme, living in the two vulnerable regions of Hungary (located in the North Eastern part of the country). The target population comprised 32 655 adults, and the health data of 20 441 successfully recruited adult persons were recorded between July 2012 and February 2016. First, subjects who had hypertension, diabetes, ischemic heart disease, chronic obstructive pulmonary disease (COPD), and/or cancer were excluded. Then, further subjects were excluded because of the lack of their BMI and/or WC data, the characteristics of these 12520 Hungarian adult subjects are shown in Additional Table 1 (see Additional file 1). Finally, because of their high BMI (≥25  kg/m^2^) scores, 7292 subjects were excluded. The final sample was formed by 5228 non-obese individuals, who were divided into two groups according to the WC. The two groups were formed based on the definition and thresholds – WC >102 cm in men and WC >88 cm in women – of Adult Treatment Panel III (ATP III), which presents the National Cholesterol Education Program’s (NCEP’s) updated recommendations for cholesterol testing and management [18]. There were 4621 persons in the group of normal WC (NWC), and 607 persons in the group of high WC (HWC) (Fig 1).

**Table 1:** Normal ranges of laboratory parameters according to ATP III/WHO

Laboratory parameters	Normal Range	Organization
Serum cholesterol	< 5.2 mmol/L	ATP III
Serum LDL cholesterol	< 2.6 mmol/L	ATP III
Serum HDL cholesterol	≥1.0 mmol/L	ATP III
Serum triglyceride	< 1.7 mmol/L	ATP III
Fasting blood glucose level	< 6.1 mmol/L	WHO

### Measurements

The HSA involved a health interview, physical examination (performed by a public health practitioner and a community nurse), and laboratory tests. The data collected and the questions applied in the HSA are shown in Additional file 2 (see Additional file 2).

Based on the definition of metabolic syndrome [18], laboratory data of blood glucose, total cholesterol, LDL cholesterol, HDL cholesterol, and triglyceride levels were used. The thresholds for laboratory parameters given by the ATP III or the WHO [19] are shown in Table 1.

The public health professionals followed the WHO STEPS (STEPwise approach to surveillance) protocol to measure the WC, that is, the measurement is made at the approximate midpoint between the lowest rib and the iliac crest [20]. BMI was defined as the body weight divided by the square of the body height, and expressed in units of kg/m^2^. Subjects with a BMI <25 kg/m^2^ were classified as non-obese, whereas those with BMI ≥25 kg/m^2^ were classified as obese according to the WHO definitions [21].

Blood pressure was measured by electronic blood pressure monitor after a five-minute rest. The classification of the American Heart Association, systolic blood pressure ≥140 mmHg regarded as *high* blood pressure, was applied [22].

### Statistical analysis

A chi-square test was performed to show the basic characteristics of the two groups. Pearson’s correlation analysis was carried out to analyze the correlation between BMI and WC. A multiple logistic regression model was used to define the association between the metabolic risk factors and abdominal obesity with adjustments for age and gender. Metabolic risk factors were used as dependent variables, and abdominal obesity was defined as an independent variable. Odds ratios (OR) and 95% confidence intervals (CI) are presented for high systolic blood pressure, high fasting blood glucose, high total cholesterol, low HDL cholesterol, high LDL cholesterol, and high triglyceride levels. A p-value lower than 0.05 was considered statistically significant. Statistical analysis was performed by using the SPSS 24.0 software.

## Results

The final sample involved 5228 non-obese subjects. Out of them, 607 subjects (11.6%) had abdominal obesity (HWC group), and there were 4621 subjects (88.4%) with no abdominal obesity (NWC group) (Table 2). Both groups involved more women than men, and the majority of the patients were young (18–29 years) or middle-aged (30–44 years) (Table 2).

**Table 2:** Characteristics of 5228 Hungarian adult subjects the data of who were recorded in the framework of the Swiss–Hungarian Cooperation Programme (2012–2016)

Characteristics	HWC group *n* (%)	NWC group *n* (%)	Total *n* (%)
WC	607 (11.6)	4621 (88.4)	5228 (100.0)
Gender***			
Male	27 (4.4)	2055 (44.5)	2082 (39.8)
Female	580 (95.6)	2566 (55.5)	3146 (60.2)
Total	607 (100.0)	4621 (100.0)	5228 (100.0)
Age***			
18–29	113 (18.6)	1852 (40.1)	1965 (37.6)
30–44	166 (27.3)	1463 (31.7)	1629 (31.2)
45–59	161 (26.5)	866 (18.7)	1027 (19.6)
60–74	111 (18.3)	323 (7.0)	434 (8.3)
≥ 75	56 (9.2)	117 (2.5)	173 (3.3)
Total	607 (100.0)	4621 (100.0)	5228 (100.0)
Systolic blood pressure***			
Normal (< 140 mmHg)	441 (74.2)	3884 (85.2)	4325 (83.9)
High (≥140 mmHg)	153 (25.8)	675 (14.8)	828 (16.1)
Total	594 (100.0)	4559 (100.0)	5153 (100.0)
Fasting blood glucose**			
Normal (< 6.1 mmol/L)	448 (91.2)	3361 (94.3)	3809 (93.9)
High (≥6.1 mmol/L)	43 (8.8)	205 (5.7)	248 (6.1)
Total	491 (100.0)	3566 (100.0)	4057 (100.0)
Total cholesterol***			
Normal (< 5.2 mmol/L)	243 (50.3)	2184 (62.5)	2427 (61.0)
High (≥5.2 mmol/L)	240 (49.7)	1313 (37.5)	1553 (39.0)
Total	483 (100.0)	3497 (100.0)	3980 (100.0)
HDL cholesterol			
Normal (≥1.0 mmol/L)	292 (95.4)	2356 (95.8)	2648 (95.7)
Low (< 1.0 mmol/L)	14 (4.6)	104 (4.2)	118 (4.3)
Total	306 (100.0)	2460 (100.0)	2766 (100.0)
LDL cholesterol			
Normal (< 2.6 mmol/L)	70 (31.4)	616 (34.3)	686 (34.0)
High (≥2.6 mmol/L)	153 (68.6)	1181 (65.7)	1334 (66.0)
Total	223 (100.0)	1797 (100.0)	2020 (100.0)
Triglyceride**			
Normal (< 1.7 mmol/L)	385 (80.2)	2966 (85.2)	3351 (84.6)
High (≥1.7 mmol/L)	95 (19.8)	514 (14.8)	609 (15.4)
Total	480 (100.0)	3480 (100.0)	3960 (100.0)

**LDL**: low-density lipoprotein; **HDL**: high-density lipoprotein

**NWC**: normal WC group with normal weight: WC ≤88 cm and BMI < 25 kg/m^2^ in females; and WC ≤102 cm and BMI < 25 kg/m^2^ in males

**HWC**: high WC group with normal weight: WC > 88 cm and BMI < 25 kg/m^2^ in females; and WC > 102 cm and BMI < 25 kg/m^2^ in males

**BMI**: body mass index; **WC**: waist circumference

**: *p* < 0.01; ***: *p* < 0.001 (Chi-square test of independence)

Some further data in Table 2 demonstrate that a significantly higher occurrence was found in the HWC group vs the NWC group of high systolic blood pressure (p<0.001), high fasting blood glucose (p=0.009), high total cholesterol level (p<0.001), and high triglyceride levels (p=0.004). The levels of HDL and LDL cholesterol were not significantly different between the two groups.

The correlation analysis suggested that WC and BMI had a significant correlation (p<0.001 both in males and females). The Pearson’s correlation coefficients between WC and BMI were 0.610 in males and 0.526 in females.

According to the results of the multiple logistic regression models, older people had significantly higher odds for developing most of the metabolic risk factors. Subjects in the age group of 30–44 years were significantly more likely to have high systolic blood pressure, high fasting blood glucose, high total cholesterol, and high LDL cholesterol levels compared to the 18–29 year age group (Table 3). This significantly increased risk of the occurrence of the above mentioned metabolic risk factors was shown in the older age groups.

**Table 3:** Odds ratios of metabolic risk factors in the Hungarian subjects with and with no abdominal obesity adjusted for age and gender (multiple logistic regression models, data collected in the framework of the Programme between 2012 and 2016)

Characteristics	High systolic blood pressure (≥140 mmHg)	High fasting blood glucose (≥6.1 mmol/L)	High total cholesterol (> 5.2 mmol/L)	Low HDL cholesterol (< 1.0 mmol/L)	High LDL cholesterol(≥2.6 mmol/L)	High triglyceride (≥1.7 mmol/L)
	OR (95% CI)	OR (95% CI)	OR (95% CI)	OR (95% CI)	OR (95% CI)	OR (95% CI)
**Age** (reference: 20–31)						
30–44	2.01 (1.55–2.60)***	2.36 (1.51–3.67)***	2.93 (2.45–3.50)***	0.94 (0.61–1.44)	2.98 (2.37–3.75)***	1.20 (0.96–1.50)
45–59	7.28 (5.71–9.28)***	5.01 (3.27–7.68)***	7.35 (6.03–8.95)***	0.53 (0.30–0.94)*	6.55 (4.89–8.78)***	1.51 (1.18–1.92)***
60–74	13.56 (10.24–17.96)***	7.78 (4.88–12.41)***	11.06 (8.43–14.50)***	0.25 (0.08–0.80)*	5.44 (3.62–8.18)***	1.78 (1.31–2.42)***
≥ 75	22.21 (15.32–32.21)***	10.22 (5.82–17.94)***	6.08 (4.23–8.75)***	1.17 (0.48–2.85)	5.37 (2.97–9.73)***	1.40 (0.88–2.24)
Gender (reference: females)	1.97 (1.65–2.35)***	1.84 (1.38–2.45)***	0.89 (0.77–1.03)	2.89 (1.90–4.37)***	0.92 (0.75–1.13)	1.79 (1.45–2.16)***
WC (reference: NWC group)	1.53 (1.20–1.94)***	1.37 (0.93–2.01)	1.02 (0.82–1.27)	2.06 (1.09–3.89)*	0.75 (0.54–1.05)	1.65 (1.27–2.16)***

**LDL** Low-density lipoprotein; **HDL** High-density lipoprotein

**NWC** Normal WC group with normal weight: WC ≤88 cm and BMI < 25 kg/m^2^ in females; and WC ≤102 cm and BMI < 25 kg/m^2^ in males

**WC**: waist circumference; **OR**: odds ratio; **CI**: confidence interval

Odds ratios were calculated using multiple logistic regression models in which the dependent variable was defined as having metabolic risk factors and it was adjusted for age and gender. The reference group was set as the females and the NWC group

*: *p* < 0.05; ***: p < 0.001

An increased risk for high triglyceride level was observed in the 45–59 and 60–74 year old age groups compared to the youngest age group. Subjects in the 45–59 and 60–74 year old age groups had significantly lower odds for low HDL cholesterol. In contrast, the risk increased in the oldest age group, although not significantly (Table 3).

Regarding gender, males had significantly higher odds for having high systolic blood pressure, high fasting blood glucose, low HDL cholesterol, and high triglyceride levels (Table 3).

Subjects in the HWC group were more likely to have some metabolic risk factors compared to those in the NWC group. If a patient had abdominal obesity, the odds for high systolic blood pressure, low HDL cholesterol, and high triglyceride levels increased significantly (Table 3). On the contrary, the risk for high LDL level was lower in the HWC group compared to the NWC group, but it was not significant. The odds of high total cholesterol level was nearly the same in the NWC and the HWC groups (Table 3).

## Discussion

In this cross-sectional study, we examined the association of abdominal obesity assessed by WC with the occurrence of metabolic risk factors in non-obese subjects. 5228 subjects were involved in our research, mostly young or middle-aged, and more females than males. Our results showed a high prevalence of abdominal obesity (11.6%) among non-obese subjects. Our study also presented a very high, positive correlation between BMI and WC, which is consistent with other studies [23, 24].

Subjects with abdominal obesity were proved to have significantly higher prevalence of high systolic blood pressure, high fasting blood glucose, high total cholesterol, and high triglyceride levels than subjects in the NWC group.

The risk for having high systolic blood pressure, high fasting blood glucose, high total cholesterol, and high LDL cholesterol levels was age-dependent, these levels were significantly higher in the age group of 30–44 years, and the risk was also significant in the older age groups. In case of high triglyceride level, an increased risk was found in the age groups 45–59 and 60–74 years. In general, as individuals grow older, the body fat content increases, especially in the abdominal region, which may be the cause of the elevated metabolic risk [25]. Therefore, screening of abdominal obesity is crucial in the elderly. Furthermore, males were significantly more affected by most of the cardiometabolic parameters, hence it would be fundamental to measure WC particularly in males. Likewise, the logistic regression model indicated that patients with abdominal obesity had significantly higher odds for high systolic blood pressure, low HDL cholesterol, and high triglyceride levels.

Our results are in line with the findings of other researchers [26, 27]. In the study of Okosun et al., WC is positively associated with having two or more components of the metabolic syndrome in three ethnic groups in Americans [26]. According to the findings of Huang, WC is a better predictor of insulin resistance [28] and a better predictor of mortality [8] than BMI. Furthermore, a strong association has also been documented between abdominal obesity, CVDs, and total mortality [27].

It is well-known that obesity is strongly related to metabolic, CV, and other diseases [29]. The health risks of abdominal obesity have already been recognized as well, although WC is still less commonly measured than BMI in the clinical practice [8]. It is of importance that some individuals with normal BMI are insulin resistant and have metabolic abnormalities, which might contribute to an abnormal fat distribution, especially abdominal obesity [30].

It is very important to measure body weight, height, and WC in a professional and reliable way, for example, during health examinations, as in self-reported health interviews, the subjects tend to underestimate their weight and/or overestimate body height [31].

Obviously, there are differences in the recommendations regarding the screening of obesity in the world. The Obesity Society Guidelines (2013) for Managing Overweight and Obesity in Adults do not recommend measuring the WC [32]. The U.S. Preventive Services Task Force (USPSTF) recommends the screening of all adults for obesity by the calculation of the BMI, although it also states that WC may be an acceptable alternative to BMI measurement in some patient subpopulations [33]. Nonetheless, Hungary has adapted the available evidence that measurement of WC is effective in the prevention of cardiometabolic disorders; thus, according to a decree [34] regarding screenings done by GPs and financed by the health insurance, measurement of WC is to be included in the physical examination over 21 years of age, and it should be repeated every 5 years. Despite the existing legal support for screenings in primary health care, the cardiometabolic preventive services are used at much lower rates than recommended for the age group of 21–64 years, and it might contribute to the extremely high CV mortality in Hungary [35]. In a study performed in a relatively large sample (3121 subjects), cardiometabolic risk has been assessed in only 44.45% of the individuals [36].

The logistic regression model also showed that the risk for elevated LDL cholesterol level was lower in patients with abdominal obesity. This is consistent with a study [37] in which it was found that high LDL cholesterol remained relatively unchanged with inreased WC. Possibly, the LDL cholesterol level was modified by cholesterol-lowering therapy, which primarily aims to decrease LDL cholesterol level (it is one of the known limitations, see below).

Using the BMI alone to identify metabolic risks, several patients with increased risk but normal BMI would be missed. To measure WC is particularly important above 30 years of age and in males, irrespective of their BMI.

The strengths of our study were the large sample size, the measurement of WC by trained health personnel, and the interpretation of the results of a new, innovative model programme in terms of screening in primary care in Hungary.

Our limitations involve that the study was not representative (selection bias), and we could not determine cause-and-effect relationships due to the cross-sectional type of this study.

Finally, not all laboratory parameters were available for each subject. In this analysis, we applied the definition of ATP III to classify the patient as having abdominal obesity (but data were not analyzed according to International Diabetes Federation (IDF) thresholds). As we did not have information on which patients had cholesterol lowering therapy, we could not exclude them from the present analysis, although this therapy might modify the measured LDL cholesterol levels and model calculations based on them.

## Conclusions

Our results suggest that screening of abdominal obesity by applying a simple, professionally performed measurement of WC might be a suitable predictor of metabolic syndrome, potentially more practical than, e.g., BMI.

The prevention of metabolic syndrome and the possible outcomes, such as CVDs and diabetes, would be especially beneficial in the Hungarian population, where CVDs are the leading causes of death, and the morbidity of T2DM is continuously increasing.

## Supplementary information

**Additional file 1: Table S1.** Characteristics of 12,520 Hungarian adult subjects – the data of who were recorded in the framework of the Swiss–Hungarian Cooperation Programme (2012–2016).

**Additional file 2.** The data collected and the questions applied in the HSA.

## Data Availability

The datasets used and/or analysed during the current study are available from the corresponding author on reasonable request.

## Abbreviations

ATP III: Adult Treatment Panel III
BMI: Body mass index
CI: 95% confidence interval
COPD: Chronic obstructive pulmonary disease
CVD: Cardiovascular disease
GPC: General practitioners’ cluster
HDL: High density lipoprotein
HSA: Health status assessment
HWC: High waist circumference
IDF: International Diabetes Federation
LDL: low density lipoprotein
NCEP: National Cholesterol Education Program
NWC: Normal waist circumference
OR: Odds ratios
SPSS: Statistical Package for the Social Sciences
T2DM: Type 2 diabetes mellitus
USPSTF: United States Preventive Services Task Force
WC: Waist circumference
WHO: World Health Organization
