# Supplementary material for: Abdominal obesity increases metabolic risk factors in non-obese adults: a Hungarian cross-sectional study
Source: BMC Public Health. 2019 Nov 15;19:1533. doi: 10.1186/s12889-019-7839-1 (PMC6858760; doi:10.1186/s12889-019-7839-1)
Supplement: Supplementary file 1 — Additional file 1: Table S1. Characteristics of 12,520 Hungarian adult subjects – the data of who were recorded in the framework of the Swiss–Hungarian Cooperation Programme (2012–2016). [file 12889_2019_7839_MOESM1_ESM.docx]

**Additional Table 1**

Characteristics of 12520 Hungarian adult subjects – the data of who were recorded in the framework of the Swiss–Hungarian Cooperation Programme (2012–2016)

| **Characteristics** | **Frequency (n)** | **Percent (%)** |
| --- | --- | --- |
| **Gender** |  |  |
| Male | 5429 | 43.4 |
| Female | 7091 | 56.6 |
| Total | 12520 | 100.0 |
| **Age** |  |  |
| 18–29 | 3142 | 25.1 |
| 30–44 | 3690 | 29.5 |
| 45–59 | 3261 | 26.0 |
| 60–74 | 1881 | 15.0 |
| ≥ 75 | 546 | 4.4 |
| Total | 12520 | 100.0 |
| **BMI** |  |  |
| normal (≤25 kg/m^2^) | 5228 | 41.8 |
| high (>25 kg/m^2^) | 7292 | 58.2 |
| Total | 12520 | 100.0 |
| **WC** |  |  |
| normal (≤88 cm in females, ≤102 cm in males) | 6778 | 54.1 |
| high (>88 cm in females, >102 cm in males) | 5742 | 45.9 |
| Total | 12520 | 100.0 |
| **Systolic blood pressure** |  |  |
| Normal (<140 mmHg) | 8754 | 71.4 |
| High (≥140 mmHg) | 3498 | 28.6 |
| Total | 12252 | 100.0 |
| **Fasting blood glucose** |  |  |
| Normal (<6.1 mmol/L) | 8563 | 87.3 |
| High (≥6.1 mmol/L) | 1250 | 12.7 |
| Total | 9813 | 100.0 |
| **Total cholesterol** |  |  |
| Normal (<5.2 mmol/L) | 4998 | 51.4 |
| High (≥5.2 mmol/L) | 4733 | 48.6 |
| Total | 9731 | 100.0 |
| **HDL cholesterol** |  |  |
| Normal (≥1.0 mmol/L) | 5384 | 80.9 |
| Low (<1.0 mmol/L) | 1270 | 19.1 |
| Total | 6654 | 100.0 |
| **LDL cholesterol** |  |  |
| Normal (<2.6 mmol/L) | 4300 | 85.3 |
| High (≥2.6 mmol/L) | 741 | 14.7 |
| Total | 5041 | 100.0 |
| **Triglyceride** |  |  |
| Normal (<1.7 mmol/L) | 8338 | 87.2 |
| High (≥1.7 mmol/L) | 1227 | 12.8 |
| Total | 9565 | 100.0 |

**LDL**: low-density lipoprotein; **HDL**: high-density lipoprotein

**BMI**: body mass index, **WC**: waist circumference
