# Supplementary material for: Abdominal obesity increases metabolic risk factors in non-obese adults: a Hungarian cross-sectional study
Source: BMC Public Health. 2019 Nov 15;19:1533. doi: 10.1186/s12889-019-7839-1 (PMC6858760; doi:10.1186/s12889-019-7839-1)
Supplement: Supplementary file 2 — Additional file 2. The data collected and the questions applied in the HSA. [file 12889_2019_7839_MOESM2_ESM.docx]

**Additional file 2**

The data collected and the questions applied in the HSA are listed below:

1. Gender, age, education, ethnicity, eligibility for prescription exemption certificate

2. Height, weight, waist circumference

3. Smoking status

4. Date of the diagnosis of hypertension

5. Blood pressure measured by standardized method

6. Date of the diagnosis of diabetes mellitus

7. Fasting serum glucose concentration measured by standardized method

8. Date of the diagnosis of liver cirrhosis

9. Knowledge on healthy diet

10. Health attitude evaluation

11. Time since last:

a. cervical cancer screening

b. breast cancer screening

c. prostatic cancer screening

d. colorectal cancer screening

e. oral cavity cancer screening

f. test of visual acuity

g. test of hearing loss

h. examination of atherosclerosis

i. measurement of body weight

j. measurement of waist circumference

k. assessment of dietary habit

l. screening for alcohol misuse

m. assessment of smoking habit

n. measurement of blood pressure

o. measurement of serum lipid parameters

p. measurement of serum glucose

q. measurement of urinary creatinine

r. measurement of urinary protein

s. assessment of family history

12. Symptoms of cirrhosis

13. How many times has been the patient referred by the GP in the last 12 months?

14. How many times has the survey participant used the services of non-medical health professionals in the last 12 months?

HSA: health status assessment

GP: general practitioner
